# New Challenges in the Monitoring, Risk Assessment, and Management of Pesticides and Biocides in the “One Health Era”

**DOI:** 10.3390/jox16010035

**Published:** 2026-02-14

**Authors:** Teresa D’Amore

**Affiliations:** 1 Department of Food Safety, Nutrition and Veterinary Public Health, Istituto Superiore di Sanità (ISS)—Italian National Institute of Health, Viale Regina Elena 299, 00161 Rome, Italy; teresa.damore@iss.it; 2 Department of Pharmacy, University of Naples, Federico II, 80131 Naples, Italy

## 1. Introduction

Pesticides and biocides remain indispensable chemicals for agriculture, food safety, public health, and industrial applications, as they safeguard crop yields, control disease vectors, and maintain high hygiene standards. However, their presence in food, feed, animals, and humans raises significant concerns for health and the environment, making their monitoring, assessment, and management a continuing public health priority [1,2].

The regulation and management of pesticides and biocides represents one of the most complex and innovation-driven fields of chemical governance. It is particularly relevant in the context of the One Health approach, which acknowledges the inherent interconnectedness of human, animal, and environmental health. Globally, a variety of frameworks exist that impose stringent requirements to ensure safety and their controlled use under specific conditions. In Europe (EU), the Regulations (EC) No. 1107/2009 (Plant Protection Products Regulation—PPPR) and No. 283/2013 concern the general conditions and requirements for the placing on the market of plant protection products; the Regulation (EU) No. 528/2012 (Biocidal Products Regulation—BPR) relates to biocidal products; and the Regulation (EC) No. 1272/2008 is pertinent to the classification, packaging, and labeling (CLP) of substances and mixtures. In the United States (US), the Federal Insecticide, Fungicide, and Rodenticide Act (FIFRA) and the Toxic Substances Control Act (TSCA), as well as the Federal Food, Drug, and Cosmetic Act (FFDCA), empower the Environmental Protection Agency (EPA) to establish tolerances—defined as maximum legally permissible levels—for pesticide residues in food. The EPA is also responsible for conducting risk assessment for the registration of pesticides. Conversely, in the EU, the responsibility of risk assessment of active substances falls under the mandates of the European Food Safety Authority (EFSA), while the European Commission and Member States take risk management decisions, including the approval of active substances and setting of maximum residue levels (MRLs) for pesticide residues in food and feed, listed in the Regulation (EC) No. 396/2005 [3,4].

Recent regulatory developments have introduced new hazard classes, including endocrine disruptors (ED), PBT/vPvB, and PMT/vPvM, which have significantly increased the complexity of data requirements for pesticides and biocides. These changes necessitate the implementation of advanced analytical strategies, mechanistic studies, and integrated risk assessments that extend beyond the scope of traditional toxicology. The identification of endocrine-disrupting properties, for example, necessitates detailed mode-of-action evidence and adverse outcome characterization, as reflected in EU criteria and US screening programs [5,6,7].

Evidently, this topic is multidisciplinary, spanning food chemistry, agricultural and biological science, toxicology, ecotoxicology, exposure science, risk assessment, and public health. It involves a globally interconnected network of actors, including researchers, academics, policy makers, authorities (e.g., EFSA, ECHA, ECDC, EPA, FDA, OECD), and others, working toward harmonized standards and coordinated assessments.

Initiatives such as “One Substance—One Assessment” in the EU and international collaborations under OECD exemplify efforts to streamline these processes. Similarly, in an effort to strength the challenges to ensure the best protection of human and environmental health, global strategies have been implemented, including the United Nations 2030 Agenda for Sustainable Development, the Farm to Fork Strategy, and the Chemicals Strategy for Sustainability [8,9].

Furthermore, the framework governing pesticides and biocides has been identified as a catalyst for scientific and technological innovation. The implementation of New Approach Methodologies (NAMs), Adverse Outcome Pathways (AOPs), in silico modeling, and integrated testing strategies is leading to a paradigm shift in risk assessment methodologies, thereby enabling the transition toward Next Generation Risk Assessment (NGRA). Programs such as the EU Partnership for the Assessment of Risks from Chemicals (PARC) and analogous global initiatives aspire to expedite the incorporation of these instruments into regulatory practice [10,11,12]. Despite the advancements, there are still obstacles that must be surmounted if the objective of attaining complete regulatory acceptance and harmonization of these approaches is to be realized.

In the context of the One Health, the monitoring, risk assessment, and management of pesticides and biocides necessitates not only adherence to evolving regulatory frameworks but also the development of innovative, science-based solutions that address emerging hazards and complex exposure scenarios. This Special Issue is devoted to the exploration of the aforementioned challenges and opportunities. The compendium comprises 15 peer-reviewed papers: ten research articles, three review articles, one perspective article, and one systematic review article. The contributions listed are noteworthy for their high quality and broad scope, covering a wide range of subjects, including analytical innovations, regulatory perspectives, food safety, toxicology, ecotoxicology, and emerging hazard classifications. Accordingly, the Special Issue offers a comprehensive overview of current progress and future directions in this field.

### 1.1. Analytical Innovations

Accurate detection of pesticide/biocide residues is the cornerstone of risk assessment and regulatory compliance. The advances in analytical chemistry have significantly improved sensitivity, specificity, and throughput for residue monitoring. High-resolution mass spectrometry (HRMS), liquid chromatography–tandem mass spectrometry (LC–MS/MS), and ion chromatography are very powerful tools for quantifying highly polar pesticides in complex matrices such as food and pollinator tissues. These methods not only meet stringent validation criteria but also enable multicenter harmonization, ensuring reproducibility across laboratories.

Beyond conventional residue analysis, innovative approaches such as biomimetic chromatography offer high-throughput screening for bioaccumulation potential and aquatic toxicity. The correlation between chromatographic hydrophobicity indices with bioconcentration factors and lethal concentration thresholds provides rapid proxies for ecotoxicological endpoints, reducing reliance on animal testing. Similarly, computational models, including Monte Carlo simulations and QSAR-based descriptors, are gaining traction as predictive tools for aquatic toxicity, offering ethical and cost-effective alternatives to traditional assays.

These analytical innovations support a paradigm shift toward integrated monitoring systems that combine chemical analytics with biological indicators. Honeybees, for instance, are increasingly recognized as bioindicators of environmental contamination, and validated methods for pesticide detection in bee matrices strengthen pollinator protection strategies. Such advancements comply with One Health principles by linking residue monitoring to environmental and human health.

### 1.2. Regulatory Perspectives and New Requirements

The European Union has established one of the most comprehensive regulatory frameworks for pesticide and biocide risk assessment. Yet, significant challenges persist. Current methodologies often fall short in addressing cumulative exposure, mixture toxicity, and low-dose effects—issues that are increasingly relevant given the complexity of real-world exposures. Recent policy developments show the need for integrated approaches that incorporate biomonitoring data, mechanistic toxicology, and environmental modeling into regulatory decision-making.

Endocrine-disrupting chemicals (EDCs) and reprotoxic substances remain at the forefront of regulatory concern. While progress has been made in hazard identification, gaps persist in implementing standardized criteria and testing protocols. The lack of formal recognition of emerging hazard classes, such as immunotoxicity, has been identified as a key area for improvement, with the necessity for expanded classification systems that reflect evolving scientific evidence being emphasized.

Another critical regulatory trend is the transition toward animal-free toxicology. Advances in in vitro systems, omics technologies, and computational modeling offer promising alternatives, but their integration into regulatory frameworks requires harmonized validation and acceptance criteria.

Furthermore, the renewal and approval processes for bioactive substances must adapt to incorporate sustainability considerations.

### 1.3. Toxicology and Hazard Classification

The toxicological research in this Special Issue evidences the complexity of pesticide-induced health effects. Systematic reviews of widely used fungicides reveal consistent cytotoxicity across mammalian cell lines, with oxidative stress and mitochondrial dysfunction emerging as central mechanisms. Apoptosis, endoplasmic reticulum stress, and genotoxicity further underscore the multifaceted nature of pesticide toxicity. Despite these findings, toxicokinetic data remain sparse, particularly with the regard to chronic exposure and cumulative effects—a gap that hampers accurate risk characterization.

Human biomonitoring studies provide compelling evidence of widespread pesticide and biocide exposure, from occupational settings to urban populations. Urinary analyses reveal stark exposure gradients, with farmworkers exhibiting the highest contamination levels, followed by para-occupational and general populations. Sociodemographic factors such as education, water sources, and protective practices significantly influence exposure risk, highlighting the interplay between behavioral and environmental determinants.

These data reinforce the urgency of refining hazard classifications to capture emerging endpoints. Immunotoxicity, neurotoxicity, and endocrine disruption demand systematic incorporation into regulatory schemes. Moreover, cumulative risk assessment frameworks must evolve to account for synergistic effects among chemical mixtures, moving beyond single-substance evaluations toward holistic exposure models.

### 1.4. Food Safety and Dietary Exposure Assessment

Food safety remains a central pillar of the One Health approach, requiring robust surveillance of active substance residues in high consumption commodities and transparent risk characterization across diverse populations. The contributions in this Special Issue demonstrate the efficacy of longitudinal monitoring, market surveillance, and probabilistic exposure modelling in elucidating chemical contaminants occurrence data, cumulative risks, and vulnerable subgroups.

Across multiple staple crops, multi residue contamination has emerged as a recurrent feature rather than an exception. Long term surveillance of tomatoes over four years—using a fully validated LC MS/MS MRM method in line with SANTE guidance—consistently detected mixtures dominated by fungicides, followed by insecticides and acaricides. The occurrence of up to five residues in a single sample shows the importance of moving beyond single substance assessments to evaluate mixture toxicity and aggregate exposure. Notably, the environmental mixture analysis (via Concentration Addition and Independent Action) returned MCR values ≥ 1, indicating that the mixture contribution to overall toxicity can exceed that of the single most toxic component, albeit only slightly in the scenarios examined. Consumer dietary risk modeling for adults and toddlers did not flag immediate health concerns, but the evidence nonetheless reinforces the need to systematically include cumulative exposure assessments when evaluating products prone to multiresidue findings.

A large-scale market surveillance of onions (*n* ≈ 5700 across 2021–2024) complements these insights with temporal and compositional dynamics. Residue free rates and EU MRL noncompliance fluctuated year to year, while fungicides consistently dominated the residue profile, with insecticides increasing over time—a shift likely reflecting evolving agronomic practices and pest pressures. Multiple residues peaked above 30% in certain years, and several active substances exceeded MRLs. Still, chronic dietary exposure modeled via EFSA PRIMo 3.1 remained well below ADI thresholds across population groups, with the highest signal (e.g., chlorpyrifos) staying firmly under critical levels even in high consumption regions. These outcomes demonstrate the value of commodity-specific surveillance over extended windows, revealing both compliance trends and the real-world relationship between occurrence, regulatory limits, and modeled health impacts.

A complementary matrix assessment of root and tuber vegetables (potatoes, carrots, celery, radishes, horseradish, ginger, onions, leeks) adds critical population-level nuance. Using validated HPLC MS/MS and GC MS/MS methods, investigators detected 33 distinct pesticides, with multiple residues present in a subset and MRL exceedances recorded in others. Acute and chronic dietary risks were quantified with hazard quotients (HQ) and hazard indices (HI), supported by Monte Carlo simulations to capture variability and uncertainty. While individual HQs remained below 1, cumulative acute exposure approached ~63% of ARfD in children (and ~51% in adults) for specific commodities (e.g., ginger, celery), whereas cumulative chronic exposure remained within acceptable bounds (max ~11% ADI in children, ~6% in adults). Vulnerable groups of population and consumption rates as major determinants of chronic risk variability.

Methodologically, these studies exemplify best practice agreement with EU guidance (method validation, PRIMo modeling) while demonstrating how cumulative risk, mixture contribution (MCR), and probabilistic methods (Monte Carlo) can be operationalized for routine surveillance.

### 1.5. Ecotoxicology and Environmental Monitoring

The environmental issues associated with the utilization of pesticides and biocides are of equal significance to those related to human health. Studies on aquatic ecosystems reveal nuanced interactions between chemical stressors and environmental variables. Glyphosate-based herbicides, for example, alter fungal sporulation patterns in streams, with species-specific responses that affect decomposition processes and nutrient cycling. Lithium, an emerging contaminant, exacerbates these effects, illustrating the complexity of multi-stressor scenarios.

Pollinators, particularly honeybees, remain sentinel species for agrochemical impacts. Advanced analytical methods now enable precise quantification of highly polar pesticides in bee matrices, supporting risk assessments that extend beyond human health to ecosystem integrity. 

Similarly, marine monitoring strategies employing biofilm mesocosms, passive samplers, and grab water analyses provide complementary insights into contaminant dynamics in coastal environments. These approaches reveal the prevalence of contaminants of emerging concern, such as UV filters and pharmaceuticals, underscoring the need for integrated surveillance systems.

Finally, bioremediation continues to be a promising strategy for mitigating environmental contamination. Microbial degradation of fluorinated pyrethroids demonstrates the potential of harnessing natural processes to detoxify persistent pollutants. The identification of cytochrome P450 enzymes in bacterial strains opens new frontiers for enzymatic remediation, offering sustainable solutions to pesticide persistence.

## 2. Conclusions and Future Perspectives

The research presented in this Special Issue advances the science and policy of pesticide and biocide assessment and management under the One Health paradigm.

Indeed, it is imperative to acknowledge that, when integrated approaches prove effective, several notable success stories emerge and illustrate how improved assessment and monitoring of both regulated products and contaminants can translate into tangible mitigation measures and more sustainable agricultural practices. For instance, the phase-out of highly hazardous organophosphates in the EU and US, following strengthened toxicological and exposure assessments, has led to measurable reductions in dietary and occupational risk. In a similar manner, strengthened ecotoxicological weight of evidence approaches, incorporated chronic toxicity endpoints, residue quantification in pollinators, and field-realistic exposure scenarios, were decisive in the EU restrictions of neonicotinoids, actions that subsequently led to the adoption of integrated pest management (IPM) strategies and stimulated the development of low-risk plant protection products. In parallel, repeated MRL exceedances detected in national residue monitoring plans have led growers to adjust pre-harvest intervals, optimize application timing, and adopt drift-reduction technologies, demonstrating how analytical evidence can directly guide agricultural practice.

Powerful take-home messages emerge:

- Integration is imperative. Analytical innovations, toxicological evaluation, and regulatory frameworks must converge to address the complexity of real-world exposures. This requires harmonized methodologies, interoperable data systems, and cross-sector collaboration.
- Knowledge gaps must be prioritized. Chronic exposure, mixture toxicity, and emerging hazard classes remain underexplored. Long-term and multigenerational studies are essential to capture cumulative effects and inform precautionary policies/mitigation measures.
- Innovation must accelerate. Predictive models, high-throughput screening tools, and omics-based assays offer pathways to reduce animal testing and enhance mechanistic understanding. Their regulatory acceptance is contingent upon the implementation of rigorous validation processes and effective stakeholder engagement strategies.
- Sustainability is non-negotiable. The transition to low-risk plant protection products, along with bioremediation strategies, links chemical management with biodiversity and climate objectives. Policy frameworks must incentivize these innovations.
- One Health is the way. In terms of future projections, the One Health approach offers a unifying lens through which to navigate these challenges.

Finally, it is evident that the future of pesticide and biocide management will depend on the seamless convergence of analytical chemistry, exposure science, mechanistic toxicology, NAM-based hazard evaluation, and responsive regulatory actions. Only when research, regulation and policy evolve jointly can we achieve the highest level of protection for entire world health under the One Health framework. The trajectory of the past decade shows that significant progress is achievable, and the most impactful advances are still to come.

## Abbreviations

The following abbreviations are used in this manuscript:

ADI	Acceptable Daily Intake
AOP	Adverse Outcome Pathway
ARfD	Acute Reference Dose
BPR	Biocidal Products Regulation
CECs	Contaminants of Emerging Concern
CLP	Classification, Labeling and Packaging
ED/EDCs	Endocrine Disruptor/Endocrine-Disrupting Chemicals
ECHA	European Chemicals Agency
ECDC	European Centre for Disease Prevention and Control
EFSA	European Food Safety Authority
EPA	Environmental Protection Agency
EU	European Union
FFDCA	Federal Food, Drug, and Cosmetic Act
FIFRA	Federal Insecticide, Fungicide, and Rodenticide Act
GC-MS/MS	Gas Chromatography Tandem Mass Spectrometry
HI	Hazard Index
HPLC-MS/MS	High-Performance Liquid Chromatography Tandem Mass Spectrometry
HQ	Hazard Quotient
HRMS	High-Resolution Mass Spectrometry
IC-HRMS	Ion Chromatography-High-Resolution Mass Spectrometry
LC-MS/MS	Liquid Chromatography Tandem Mass Spectrometry
MCR	Mixture Contribution Ratio
MRL	Maximum Residue Level
NAMs	New Approach Methodologies
NGRA	Next Generation Risk Assessment
OECD	Organization for Economic Co-operation and Development
PARC	Partnership for the Assessment of Risks from Chemicals
PBT	Persistent, Bioaccumulative and Toxic
PMT	Persistent, Mobile and Toxic
PPPR	Plant Protection Products Regulation
PRIMo	Pesticide Residue Intake Model
QSAR	Quantitative Structure
SANTE	Directorate-General for Health and Food Safety (European Commission)
TSCA	Toxic Substances Control Act
vPvB	very Persistent and very Bioaccumulative
vPvM	very Persistent and very Mobile
